# Network Pharmacology Study on Molecular Mechanisms of Zhishi Xiebai Guizhi Decoction in the Treatment of Coronary Heart Disease

**DOI:** 10.1155/2021/3574321

**Published:** 2021-12-20

**Authors:** Jin Gao, Yujing Pan, Yuxi Zhao, Haoyang Li, Zishuo Mi, Hao Chen, Xiaodong Tan

**Affiliations:** ^1^School of Integrated Chinese and Western Medicine, Nanjing 210023, China; ^2^School of Acupuncture and Massage, Nanjing University of Chinese Medicine, Nanjing 210023, China; ^3^College of Pharmacy, Nanjing University of Chinese Medicine, Nanjing 210023, China; ^4^First Clinical Medical College, Nanjing University of Chinese Medicine, Nanjing 210023, China; ^5^School of Nursing, Nanjing University of Chinese Medicine, Nanjing 210023, China; ^6^Department of Cardiovascular Medicine, Hospital of Traditional Chinese Medicine of Wuxi City, Wuxi 214000, China

## Abstract

**Background:**

Coronary heart disease is characterized by the formation of arterial plaque. If not taken seriously, it will cause serious consequences such as myocardial infarction and heart failure. Zhishi Xiebai Guizhi Decoction first appeared in “Synopsis of Prescriptions of the Golden Chamber” and is a representative prescription for the treatment of coronary heart disease. This study aims to explain the mechanism of Zhishi Xiebai Guizhi Decoction in the treatment of coronary heart disease through network pharmacology and clinical trials.

**Methods:**

We first identified the core compounds of Zhishi Xiebai Guizhi Decoction and their potential targets through TCMSP. Then, We analyzed the molecular targets of Zhishi Xiebai Guizhi Decoction in coronary heart disease with OMIM and GeneCards databases. After the common targets were screened out, we manage to figure out the pathways of these target genes through STRING. Finally, we verify the treatment results in clinical trials.

**Results:**

Through network pharmacology analysis, we discovered that several core compounds of Zhishi Xiebai Guizhi Decoction have anti-inflammatory effects and are of great significance to treatment of cardiovascular diseases. The mechanism may be closely related to PPAR*γ*, inflammation, TNF signaling pathway, AMPK signaling pathway, and PI3K-Akt signaling pathway. Clinical trials have also proved the key role of inflammation.

**Conclusions:**

Zhishi Xiebai Guizhi Decoction may play a role in treating coronary heart disease by activating PPAR*γ*. TNF signaling pathway, AMPK signaling pathway, and PI3K-Akt signaling pathway are potential mechanisms as well. The application of network pharmacology can provide a novel method for the research of Chinese herbal medicine. We hope that Zhishi Xiebai Guizhi Decoction will be recognized as a complementary or alternative treatment for coronary heart disease.

## 1. Introduction

Coronary heart disease (CHD) is characterized by formation of arterial plaques which are mainly comprised of lipids, calcium, and inflammatory cells [1]. These plaques narrow the lumen of coronary arteries leading to episodic or persistent angina. Rupture of these plaques results in the appearance of thrombus, which, brought about by cessation of blood flow, causes myocardial infarct and death [2]. CHD is one of the leading causes of death worldwide [3, 4]. The increasing number of CHD patients will lay a heavy economic burden on society [5]. Currently, the drugs commonly used in clinic to treat coronary heart disease are statins, nitrate esters, etc., with which the residual risk of cardiovascular events cannot be completely eliminated after treatment [6, 7]. Given this, many doctors have been seeking alternative medicines to treat CHD. Traditional Chinese medicine (TCM), as a type of alternative drug, displays the merits of low side effects and less irritation to the gastrointestinal tract [8, 9]. It has been demonstrated that TCM works an outstanding clinical effect when treating CHD [10]. Zhishi Xiebai Guizhi Decoction is effective. This prescription contains Aurantii Fructus Immaturus (“ZhiShi” in Chinese, ZS), Allium Azureum Ledeb. (“XieBai” in Chinese, XB), Cinnamomi Ramulus (“GuiZhi” in Chinese, GZ), Trichosanthes Kirilowii Maxim (“GuaLou” in Chinese, GL), and Magnolia Officinalis Rehd Et Wils. (“HouPo” in Chinese, HP). Multidrug compatibility is regarded as the essence of TCM theory [11]. However, due to the complex components and numerous targets involved, fully elucidating its mechanism using traditional methods is challenging. Therefore, it is necessary to reveal the potential mechanism of Zhishi Xiebai Guizhi Decoction in the treatment of CHD at the systemic level.

With the continuous innovation and development of systems biology and computer technology, the network pharmacology has been confirmed as a feasible choice to explicate the substance composition and molecular mechanism of TCM effectively and systemically [12, 13]. In 2008, Hopkins proposed the concept of network pharmacology [14]. Because network pharmacology can provide a full or partial understanding of the principles of network theory and systems biology, it has been considered the next paradigm in drug discovery. In addition, the network pharmacology approach has been used to study “compound-proteins/genes-disease” pathways, which are capable of describing complexities among biological systems, drugs, and diseases from a network perspective, sharing a similar holistic philosophy as TCM [15]. The application of systems biology methods to study the pharmacological effects, mechanism of action, and safety of TCM is of great significance to modern research and development of TCM. Thus, a new interdisciplinary method termed TCM network pharmacology has been proposed, which has initiated a new research paradigm for transforming TCM from an experience-based to evidence-based medicine. Furthermore, with recent advances in molecular biology and genomic technologies, an increasing amount of data has become available [16], for example, TCMSP [17], STRING [18], OMIM [19], and DisGeNET [20].

**Figure 1:**
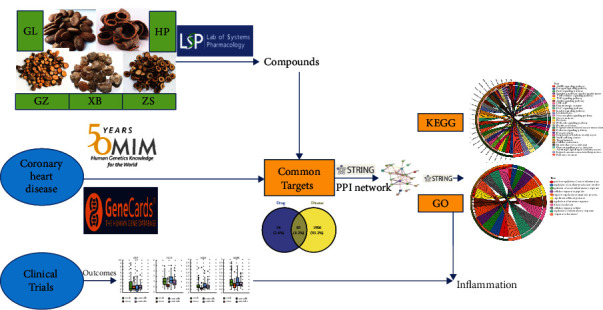
The whole framework based on an integration strategy of network pharmacology.

In this study, we used network pharmacology to predict the potential mechanism of Zhishi Xiebai Guizhi Decoction in the treatment of CHD. The workflow is displayed in Figure 1.

## 2. Methods

### 2.1. Screening the Chemical Components of Zhishi Xiebai Guizhi Decoction and Predicting the Component-Targets

The chemical ingredients of Zhishi Xiebai Guizhi Decoction were screened from TCMSP (http://lsp.nwu.edu.cn/tcmsp.php). Based on a previously reported model, we screened the various compounds in Zhishi Xiebai Guizhi Decoction according to their pharmacokinetic absorption, distribution, metabolism, and excretion, which is known as ADME process. TCMSP database details the ADME parameters of each component, including oral bioavailability (OB), druglikeness (DL), and blood-brain barrier (BBB). Ingredients meeting the demands of both OB ≥ 30% and DL ≥ 0.18 were selected to find the effective components of this prescription [21]. OB represents the oral availability of pharmaceutical ingredients, and DL refers to the similarity between a component and a known drug. Subsequently, the components in the prescription were selected (Table 1).

### 2.2. Predicting the Target Proteins of the Selected Compounds

**Figure 2:**
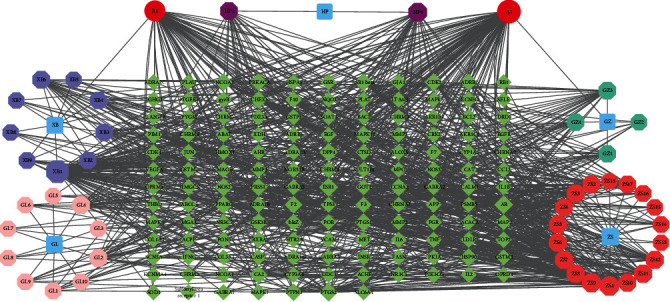
The network diagram of the compound and the target protein.

All the active ingredients were input into the TCMSP database to obtain their known targets, and the Cytoscape3.8.2 tool was used to draw a network diagram of the compound and the target protein (Figure 2).

The blue nodes represent Zhishi, Xiebai, Guizhi, Gualou, and Houpo. The red nodes represent the compounds shared by Guizhi and Xiebai. The dark purple nodes represent the compounds of Houpo. The orange nodes represent the compounds of Zhishi. The light purple nodes represent the compounds of Xiebai. The pink nodes represent the compounds of Gualou. The yellow nodes represent the compounds of Guizhi. The green nodes represent the targets related to Zhishi Xiebai Guizhi Decoction.

### 2.3. Seeking Out Disease-Related Targets

With “coronary heart disease” as the keywords, OMIM (https://www.omim.prg/) and GeneCards (https://www.genecards.org/) were used to search and screen the known disease-targets for the subsequent study, and the repeated targets in the search results were discarded. UniProt knowledge base [22, 23] (https://www.uniprot.org/) was used to get the standard targets' names with the organism selected as “Homo sapiens.”

### 2.4. Searching for Common Targets and Key Targets of Zhishi Xiebai Guizhi Decoction and CHD

**Figure 3:**
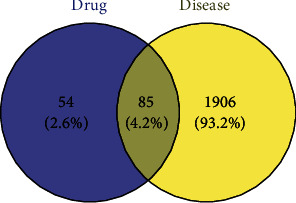
Venn diagram of targets of Zhishi Xiebai Guizhi Decoction in treating coronary heart disease.

The common targets of drug and disease were found, and a Venn diagram was drawn (Figure 3).

**Figure 4:**
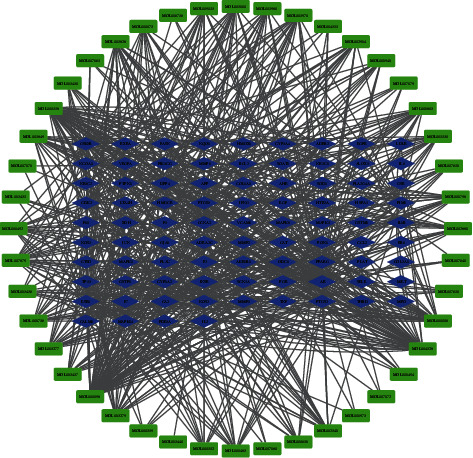
Network diagram of intersection targets of Zhishi Xiebai Guizhi Decoction in the treatment of coronary heart disease.

The obtained intersection target was used as the drug effect target, and Cytoscape3.8.2 was employed to construct the drug effect target-component interaction network (Figure 4). The network was analyzed to get its degree value and get the key drug effect target (Table 2).

The green nodes represent the compounds of Zhishi Xiebai Guizhi Decoction. The blue nodes represent the key targets related to Zhishi Xiebai Guizhi Decoction.

### 2.5. Construction of the Protein-Protein Interaction Network

**Figure 5:**
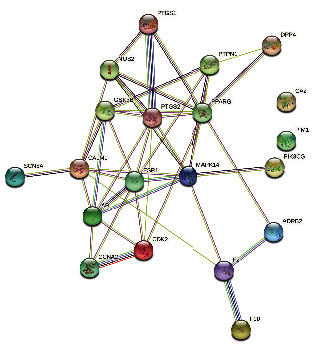
The protein-protein interaction network of Zhishi Xiebai Guizhi Decoction in the treatment of coronary heart disease.

Using the STRING (Search Tool for the Retrieval of Interacting Gene/Proteins) database containing known and predicted PPIs [24], we constructed a protein-protein interaction (PPI) network of potential target genes of Zhishi Xiebai Guizhi Decoction in CHD (Figure 5).

### 2.6. Enrichment Analysis

To identify the biological process and signaling pathways in which the main hub target genes are involved, Database for Annotation, Visualization, and Integrated Discovery (David) were used for pathway enrichment analysis. The target genes of Zhishi Xiebai Guizhi Decoction in CHD were input into David for Gene Ontology (GO) biological process analysis and Kyoto Encyclopedia of Genes and Genomes (KEGG) pathway analysis. GO biological processes with *P* ≤ 0.01 and KEGG pathways with*P* ≤ 0.01 were considered to be significantly enriched.

### 2.7. Clinical Index Changes of Zhishi Xiebai Guizhi Decoction in Treating Coronary Heart Disease Patients

A total of 176 patients with coronary heart disease were included in the clinical study. According to the random number table, the enrolled patients were divided into control group (88 cases) and the test group (88 cases). During the treatment, 7 cases were dropped from the two groups, and final effective cases were 81 cases in each group. This study was approved by the Ethics Committee of Wuxi Hospital of Traditional Chinese Medicine and registered in the Chinese Clinical Trial Registration Center (ethics number: 2018022736, registration number: ChiCTR1800019814). Before entering the group, patients and their family members were informed of all the research content and interests, were fully aware of them, and signed an informed consent form on the premise of voluntary participation. The diagnostic criteria for patients with coronary heart disease enrolled in this trial were based on the “2013 ESC guidelines on the management of stable coronary artery disease: the task force on the management of stable coronary artery disease of the European Society of Cardiology [25],” and the most diagnosed patients were patients with stable coronary artery disease. After admission, both groups were given standardized treatment for stable coronary heart disease. The test group was treated with Zhishi Xiebai Guizhi Decoction on the basis of the control group. Both groups were treated for 2 months and finally got inflammations such as Neutrophil to lymphocyte ratio (NLR), Monocyte of lymphocyte ratio (MLR), Monocyte to high-density lipoprotein ratio (MHR), and C-reaction protein (CRP) factor level changes, and preliminary exploration of the mechanism of the prescription on the inflammatory response provided a clinical basis for the later confirmation of its molecular mechanism in vitro and in vivo.

## 3. Results

### 3.1. Identification of Targets of Zhishi Xiebai Guizhi Decoction and CHD in Various Databases

The database retrieved 139 relevant targets of the active ingredient, and the active ingredient-target interaction network was constructed using Cytoscape 3.8.2 (Figure 2). Through keyword search, 1991 related targets of coronary heart disease were obtained in GeneCards database and OMIM database. The Venny diagram constructs the intersection of active ingredient-target and disease-target. A total of 85 intersection targets are used for subsequent network pharmacological analysis.

### 3.2. Seeking Key Targets and Built PPI Networks

The obtained intersection target was used as the drug effect target, and Cytoscape 3.8.2 was used to construct the drug effect target-component interaction network (Figure 4). The network was analyzed to get its degree value. The top 20 pharmacodynamic targets with degree value include estrogen receptor (ESR1), androgen receptor (AR), prostaglandin G/H synthase 2(PTGS2), and peroxisome proliferator activated receptor (PPARG) (Table 2).

### 3.3. Enrichment Analysis by GO and KEGG

**Figure 6:**
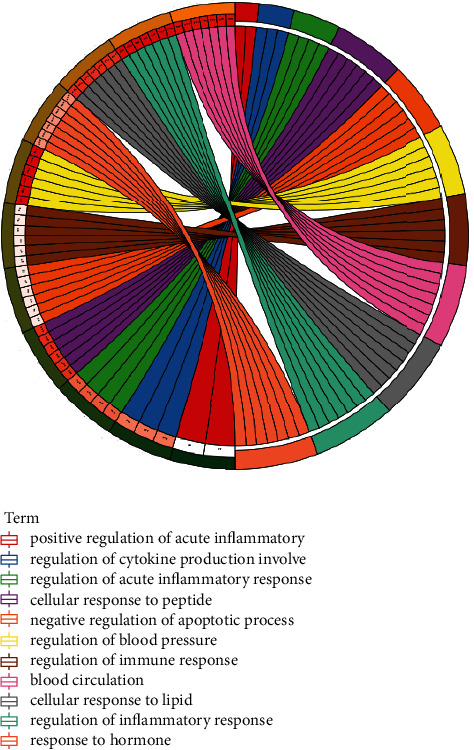
GO biological process enrichment analysis.

According to *P* value, the important items of BP of GO analysis were regulation of blood pressure, regulation of inflammatory response, blood circulation, cellular response to lipid, cellular response to peptide, response to hormone, negative regulation of apoptotic process, regulation of immune response, regulation of acute inflammatory response, regulation of cytokine production involved in inflammatory response, and positive regulation of acute inflammatory response (Figure 6). The results showed that Zhishi Xiebai Guizhi Decoction is closely related to inflammatory reaction in the treatment of CHD.

**Figure 7:**
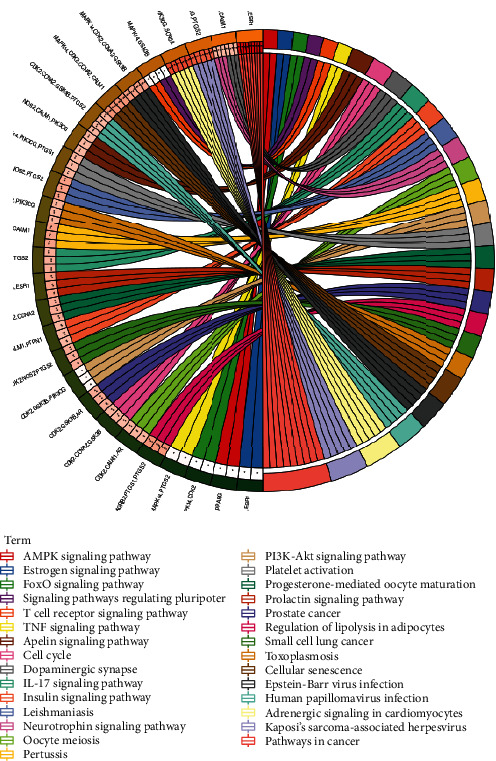
KEGG enrichment analysis.

According to the *P* value, a total of 29 pathways were screened by KEGG analysis, including pathways in cancer, adrenergic signaling in cardiomyocytes, IL-17 signaling pathway, T cell receptor signaling pathway, PI3K-Akt signaling pathway, and AMPK signaling pathway (Figure 7).

### 3.4. Clinical Trial Results

**Figure 8:**
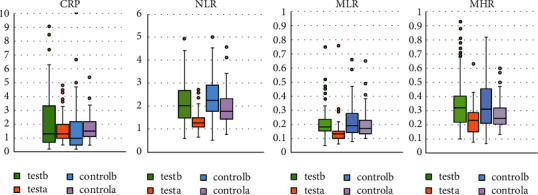
Clinical trial results.

The CRP of the two groups of patients before and after treatment showed a skewed distribution, so the median (interquartile range) was used to describe the difference, and nonparametric tests were used to compare the differences (Figure 8). The comparison of CRP in the two groups before and after treatment was statistically significant (*P* < 0.05), and the CRP levels in both the test group and the control group decreased after treatment. After rank sum test, there was no statistically significant difference between the two groups before treatment (*P* > 0.05), and there was no significant difference in CRP between the two groups after treatment (*P* > 0.05) (Table 3).

  Teseb = before treatment in the test group; Testa = after treatment in the test group
  Controlb = before treatment in the control group; Controla = after treatment in the control group.

The NLR of the two groups of patients before and after treatment showed a skewed distribution, so the median (interquartile range) was used to describe the difference, and the difference was compared with nonparametric tests (Table 4). The comparison of NLR in the two groups before and after treatment was statistically significant (*P* < 0.05), and the NLR levels in both the test group and the control group decreased after treatment. After the rank sum test, there was no statistical difference between the two groups before treatment (*P* > 0.05), and the difference in NLR between the two groups after treatment was statistically significant (*P* < 0.05).

The MLR of the two groups of patients before and after treatment showed a skewed distribution, so the median (interquartile range) was used to describe the difference, and the difference was compared with nonparametric tests (Table 5). The comparison of MLR in the two groups before and after treatment was statistically significant (*P* < 0.05), and the MLR levels in both the test group and the control group decreased after treatment. After the rank sum test, there was no statistical difference between the two groups before treatment (*P* > 0.05), and the difference in MLR between the two groups after treatment was statistically significant (*P* < 0.05).

The MHR of the two groups of patients before and after treatment showed a skewed distribution, so the median (interquartile range) was used to describe the difference, and the difference was compared with nonparametric tests (Table 6). The comparison of MHR in the two groups before and after treatment was statistically significant (*P* < 0.05), and the MHR levels in both the test group and the control group decreased after treatment. After the rank sum test, there was no statistical difference between the two groups before treatment (*P* > 0.05), and the difference in MHR between the two groups after treatment was statistically significant (*P* < 0.05).

## 4. Discussion

### 4.1. Summary of Findings

Coronary heart disease is a common cardiovascular disease, caused by coronary atherosclerosis. If not taken seriously, it will cause serious consequences such as myocardial infarction and heart failure [26]. In our study, Zhishi Xiebai Guizhi Decoction was used to treat coronary heart disease. However, illuminating the complex mechanisms of Zhishi Xiebai Guizhi Decoction in the treatment of CHD using traditional methods is challenging. Therefore, the integration of network pharmacology is essential sense based on big data bioinformatics into the study of the molecular mechanisms of TCM in the treatment of diseases [27, 28]. In the present study, network pharmacology was used to explore the material basis and molecular mechanism of Zhishi Xiebai Guizhi Decoction for treatment of CHD.

From the network of herbs, natural compounds, and targets, we found the core compounds of this prescription were quercetin, naringenin, luteolin, (+)-catechin, hesperetin, etc. Quercetin, a flavonoid, is one of the polyphenols characterized as the compounds containing large multiples of phenol structural units [29]. It can reduce blood pressure and promote angiogenesis through anti-inflammatory, antioxidant, immune, and other ways [30]. It is a potential protector of coronary heart disease, cancers, and inflammatory bowel disease [31]. It exhibits significant heart related benefits as inhibition of LDL oxidation, endothelium-independent vasodilator effects, and other inflammatory effects [32]. Naringenin has the functions of lowering lipid, anti-inflammatory, antiallergic, antithrombotic effects, and promoting atherosclerosis regression [33–35]. Luteolin administration improved cardiac function, attenuated the inflammatory response, alleviated mitochondrial injury, decreased oxidative stress, inhibited cardiac apoptosis, and enhanced autophagy [36, 37]. Studies have shown that it can attenuate isoproterenol-induced myocardial injury and fibrosis in mice [38].

85 common targets were found for drugs and diseases, which might be targets for this prescription when treating CHD. Based on the topological analysis, we further found the 20 critical targets from the 85 common targets for subsequent study, including estrogen receptor (ESR1), androgen receptor (AR), prostaglandin G/H synthase 2(PTGS2), and peroxisome proliferator activated receptor gamma (PPARG). A number of studies also provide evidence for an inhibitory role of PPAR*γ* in atherosclerosis and that it may exert atheroprotective effects [39]. The human PPAR*γ* gene is located on chromosome 3 at position 3p25 and gives rise to three different mRNAs isoforms, *γ*1, *γ*2, and *γ*3. Among them, PPAR*γ*3 is predominantly expressed in macrophages, the large intestine, and adipose tissue [40]. The pleiotropic effects of PPARs show the potential of this drug class in terms of treating atherosclerotic disease in the future [41–43], including their ability to decrease thrombosis, cell recruitment, cell activation, foam cell formation, and inflammatory responses, and their concurrent ability to improve plaque stability, endothelial function, endothelial progenitor cell biology, and C efflux. In human atherosclerotic lesions, PPAR*γ* activation has been reported to promote differentiation of proatherogenic M1 macrophages into an alternative anti-inflammatory phenotype, M2, which could protect against the development of atherosclerosis. There is accumulating evidence suggesting that activated PPAR has powerful antiatherosclerotic properties, which not only directly affects the blood vessel wall but also indirectly affects systemic inflammation [42]. A combination of our GO analysis, clinical trials, and other modern studies has confirmed the important role of inflammation in CHD. KEGG enrichment analysis shows the important position of AMPK, TNF, and PI3K-Akt signaling pathway in CHD [44–46]. PPAR*γ* plays a vital role in these pathways.

### 4.2. Implication for Clinical Trial

As we all know, inflammatory is of great essence in the pathogenesis of CHD [2]. Among the various inflammatory factors, besides the CRP that has been widely used in clinical practice, it has been generally recognized. In addition, clinical studies have confirmed that white blood cells and their subtypes are closely related to cardiovascular disease caused by atherosclerosis [47]. White blood cells are an important marker of inflammation. In recent years, experts have integrated various subtypes and recently proposed three indicators: Monocyte to lymphocyte ratio (MLR), Neutrophil to lymphocyte ratio (NLR), and Monocyte to high-density lipoprotein ratio (MHR) [48–52]. They can all be regarded as a kind of inflammatory markers and are related to coronary heart disease [53]. Almost all the compounds in Zhishi Xiebai Guizhi Decoction have anti-inflammatory effects, so we speculate that Zhishi Xiebai Guizhi Decoction treats CHD through inflammation.

### 4.3. Limitations

The key targets and/or pathways found in network pharmacology have not been verified in clinical trials, but NLR, NHR, and MHR can all be regarded as a kind of inflammatory markers and are related to coronary heart disease. The key targets and pathways also play an important role in inflammation. In the future, we need to verify our conjecture through animal experiments.

## 5. Conclusions

This study combined network pharmacology and clinical trials to explore the mechanism of Zhishi Xiebai Guizhi Decoction in the treatment of CHD. The results showed that Zhishi Xiebai Guizhi Decoction may exert antiatherosclerosis effect through PPAR*γ*. In addition, TNF, AMPK, and PI3K-Akt signaling pathway may also be its potential mechanisms. We hope that computer biology can provide a method for the modern research of Chinese medicine, and Zhishi Xiebai Guizhi Decoction can be recognized as a complementary or alternative treatment for CHD.

## Figures and Tables

**Table 1:** Effective components of Zhishi Xiebai Guizhi Decoction that meet the demands of both OB ≥ 30% and DL ≥ 0.18 were obtained from TCMSP.

Drug	ID	Components	OB%	DL

GL	MOL001494	Mandenol	41.9962	0.19321
	MOL002881	Diosmetin	31.13795	0.27442
	MOL004355	Spinasterol	42.97937	0.75534
	MOL005530	Hydroxygenkwanin	36.467	0.27206
	MOL006756	Schottenol	37.42312	0.75067
	MOL007165	10*α*-cucurbita-5,24-diene-3*β*-ol	44.01594	0.7445
	MOL007171	5-Dehydrokarounidiol	30.22665	0.7703
	MOL007172	7-Oxo-dihydrokaro-unidiol	36.85021	0.75387
	MOL007175	Karounidiol 3-o-benzoate	43.99061	0.49505
	MOL007179	Linolenic acid ethyl ester	46.10096	0.19694
	MOL007180	Vitamin-e	32.28643	0.69563

HP	MOL005970	Eucalyptol	60.62476	0.32159
	MOL005980	Neohesperidin	57.44074	0.27085

GZ	MOL000073	Ent-epicatechin	48.95984	0.24162
	MOL000358	Beta-sitosterol	36.91391	0.75123
	MOL000359	Sitosterol	36.91391	0.7512
	MOL000492	(+)-catechin	54.82643	0.24164
	MOL001736	(−)-Taxifolin	60.50622	0.27342

XB	MOL000098	Quercetin	46.43335	0.27525
	MOL000332	*n*-coumaroyltyramine	85.62883	0.20287
	MOL000358	Beta-sitosterol	36.91391	0.75123
	MOL000483	(Z)-3-(4-Hydroxy-3-methoxy-phenyl)-N-[2-(4-hydroxyphenyl) ethyl] acrylamide	118.3477	0.26399
	MOL000631	Coumaroyltyramine	112.9016	0.20234
	MOL001973	Sitosteryl acetate	40.38964	0.85102
	MOL002341	Hesperetin	70.31209	0.27252
	MOL004328	Naringenin	59.2939	0.21128
	MOL007640	Macrostemonoside e_qt	35.259	0.87216
	MOL007650	PGA (sup 1)	43.98251	0.25437
	MOL007651	Prostaglandin B1	40.20777	0.25384

ZS	MOL000006	Luteolin	36.16263	0.24552
	MOL001798	Neohesperidin_qt	71.16886	0.27085
	MOL001803	Sinensetin	50.55685	0.44634
	MOL001941	Ammidin	34.54856	0.22355
	MOL002914	Eriodyctiol (flavanone)	41.35043	0.2436
	MOL004328	Naringenin	59.2939	0.21128
	MOL005100	5,7-Dihydroxy-2-(3-hydroxy-4-methoxyphenyl) chroman-4-one	47.73644	0.27226
	MOL005828	Nobiletin	61.66944	0.51652
	MOL005849	Didymin	38.55139	0.23908
	MOL007879	Tetramethoxyluteolin	43.68476	0.37009
	MOL009053	4-[(2S,3R)-5-[(E)-3-Hydroxyprop-1-enyl]-7-methoxy-3-methylol-2,3-dihydrobenzofuran-2-yl]-2-methoxy-phenol	50.75514	0.3948
	MOL013276	Poncirin	36.54601	0.74202
	MOL013277	Isosinensetin	51.15169	0.44149
	MOL013279	5,7,4′-Trimethylapigenin	39.83272	0.29636
	MOL013352	Obacunone	43.28625	0.76724
	MOL013428	Isosakuranetin-7-rutinoside	41.24013	0.71616
	MOL013430	Prangenin	43.59734	0.29428
	MOL013433	Prangenin hydrate	72.63401	0.28863
	MOL013435	Poncimarin	63.62093	0.34942
	MOL013436	Isoponcimarin	63.2776	0.31316
	MOL013437	6-Methoxy aurapten	31.23777	0.3008
	MOL013440	Citrusin B	40.79717	0.71331

**Table 2:** Top 20 targets of Zhishi Xiebai Guizhi Decoction in the treatment of coronary heart disease.

UniP-ID	Protein names	Degree

P03372	Estrogen receptor (ESR1)	41
P10275	Androgen receptor (AR)	40
P35354	Prostaglandin G/H synthase 2 (PTGS2)	35
P37231	Peroxisome proliferator activated receptor gamma (PPARG)	32
P27487	Dipeptidyl peptidase IV (DPP4)	32
P35228	Nitric oxide synthase, inducible (NOS2)	30
P23219	Prostaglandin G/H synthase 1 (PTGS1)	28
P49841	Glycogen synthase kinase-3 beta (GSK3B)	28
Q16539	Mitogen-activated protein kinase 14 (MAPK14)	27
P24941	Cell division protein kinase 2 (CDK2)	27
P20248	Cyclin-A2 (CCNA2)	26
P00918	Carbonic anhydrase II (CA2)	26
P18031	mRNA of protein-tyrosine phosphatase, nonreceptor type 1 (PTPN1)	23
P11309	Protooncogene serine/threonine-protein kinase Pim-1 (PIM1)	23
P00734	Thrombin (F2)	22
P0DP23	Calmodulin (CALM1)	17
P48736	Phosphatidylinositol-4,5-bisphosphate 3-kinase catalytic subunit, gamma isoform (PIK3CG)	15
Q14524	Sodium channel protein type 5 subunit alpha (SCN5A)	14
P07550	Beta-2 adrenergic receptor (ADRB2)	14
P00742	Coagulation factor Xa (F10)	9

**Table 3:** Comparison of CRP between two groups (median (interquartile)).

CRP	Before treatment	After treatment	*Z* value	*P* value

Test group	1.3 (2.65)	1.3 (1.05)	−2.979	0.003
Control group	1 (1.7)	1.5 (1.1)	−3.498	≤0.001
*Z* value	−1.447	−1.590		
*P* value	0.148	0.112		

**Table 4:** Comparison of NLR between two groups (median (interquartile)).

NLR	Before treatment	After treatment	*Z* value	*P* value

Test group	2.02 (1.19)	1.27 (0.4)	−7.751	≤0.001
Control group	2.26 (1.14)	1.76 (0.89)	−6.151	≤0.001
*Z* value	−1.603	−6.844		
*P* value	0.109	≤0.001		

**Table 5:** Comparison of MLR between two groups (median (interquartile)).

MLR	Before treatment	After treatment	*Z* value	*P* value

Test group	0.18 (0.09)	0.13 (0.05)	−7.568	≤0.001
Control group	0.19 (0.14)	0.17 (0.10)	−2.935	0.003
*Z* value	−0.59	−5.061		
*P* value	0.555	≤0.001		

**Table 6:** Comparison of MHR between two groups (median (interquartile)).

MHR	Before treatment	After treatment	*Z* value	*P* value

Test group	0.32 (0.19)	0.23 (0.14)	−6.796	≤0.001
Control group	0.31 (0.25)	0.25 (0.12)	−4.066	≤0.001
*Z* value	−0.039	−2.686		
*P* value	0.969	0.007		

## Data Availability

The data used to support the findings of this study are available from the corresponding author upon request.
